# Differential Expression Profiles and Function Predictions for tRFs & tiRNAs in Skin Injury Induced by Ultraviolet Irradiation

**DOI:** 10.3389/fcell.2021.707572

**Published:** 2021-08-10

**Authors:** Yuan Fang, Yang Liu, Yu Yan, Yiyu Shen, Zenan Li, Xu Li, Yufang Zhang, Zhigang Xue, Cong Peng, Xiang Chen, Ke Cao, Jianda Zhou

**Affiliations:** ^1^Department of Plastic Surgery, The Third Xiangya Hospital, Central South University, Changsha, China; ^2^Department of Nephrology, The Second Xiangya Hospital, Central South University, Changsha, China; ^3^Anyang Tumor Hospital, The Fourth Affiliated Hospital of Henan University of Science and Technology, Anyang, China; ^4^Tongji Hospital, School of Medicine, Tongji University, Shanghai, China; ^5^Hunan Engineering Research Center of Skin Health and Disease, Changsha, China; ^6^Department of Clinical Pharmacology, Xiangya Hospital, Central South University, Changsha, China; ^7^Department of Dermatology, Xiangya Hospital, Central South University, Changsha, China; ^8^Hunan Key Laboratory of Skin Cancer and Psoriasis, Changsha, China

**Keywords:** ultraviolet irradiation, tRF & tiRNA, skin injury, sequencing, bioinformatics

## Abstract

Ultraviolet (UV) radiation is a major environmental factor contributing skin damage. As UV exposure is inevitable, it is necessary to pay attention to the underlying molecular mechanisms of UV-induced skin damage to develop effective therapies. tRNA-derived stress-induced RNAs (tiRNAs) and tRNA-derived fragments (tRFs) are tRNA-derived small RNAs (tsRNAs) that are a novel class of short, non-coding RNAs. However, the functions behind tRFs & tiRNAs in UV-induced skin injury are not yet clear. Firstly, the animal model of ultraviolet irradiation induced skin damage was established. Then the skin samples were preserved for the follow-up experiment. Sequencing was used to screen expression profiles and predict target genes. Compared with normal skin, a total of 31 differentially expressed tRFs & tiRNAs were screened. Among these, 10 tRFs & tiRNAs were shown to be significantly different in expression levels, where there were 4 up-regulated and 6 down-regulated target genes. Bioinformatics analyses revealed potential up-regulated tsRNAs (tRF-Val-AAC-012, tRF-Pro-AGG-012, tRF-Val-CAC-018, tRF-Val-AAC-031) and down-regulated tsRNAs (tRF-Arg-CCT-002, tRF-Trp-TCA-001, tiRNA-Ser-GCT-001, tRF-Gly-CCC-019, tRF-Ala-TGC-001, tRF-Ala-TGC-002). In summary, it was speculated that tRF-Gly-CCC-019 plays an important role in acute skin injury induced by UVB radiation by regulating the ras-related C3 botulinum toxin substrate 1 (Rac1) gene in the WNT signaling pathway. This study provides new insights into the mechanisms and therapeutic targets of UV-induced skin injury.

## Introduction

Ultraviolet light A (UVA) and Ultraviolet light B (UVB) radiation in sunlight causes acute damage including a sunburn or chronic cumulative damage such as photoaging and skin cancer posing a potential hazard to human health (Wolf et al., 2019). UVB (290–320 nm) has a short wavelength and high energy and mainly acts on the epidermis where keratinocytes are targeted. UVB induces adverse reactions such as skin erythema, edema, blisters, skin pigmentation, abnormal growths, light aging, and non-melanoma skin cancer (Hsu et al., 2020; Son et al., 2020; Sun et al., 2021). The body protects the skin using a variety of protective measures, such as apoptosis, inflammation and combating cells that may potentially be cancerous.

A transporter RNA (tRNA) is a connector molecule involved in decoding mRNAs and translating proteins. Recent studies have shown that tRNAs can also serve as a main source for small non-coding RNAs (sncRNAs) with unique and diverse functions (Akiyama et al., 2020). These tRNA-derived ncRNAs are not products of random degradation but are produced by precise biogenic processes (Martinez et al., 2017). The ncRNAs from tRNAs can be roughly divided into two aspects: tiRNAs (tRNA-derived stress-induced RNAs) and tRFs (tRNA derived fragments) that implement their specific molecular size, nucleotide composition, physiological function, and biogenesis (Zhu et al., 2019; Lakshmanan et al., 2021). The length of tRFs is about 16–28 nt and they are derived from mature tRNAs or precursor tRNAs. In addition, tRFs & tiRNAs are associated with a variety of pathological conditions such as cancer, neurodegenerative diseases and inherited metabolic diseases (Hanada et al., 2013; Zhang et al., 2020; Wang et al., 2021). Studies have shown that tRFs & tiRNAs in sperm represent a class of epigenetic factors that mediate inheritance of diet-induced metabolic diseases to offspring (Chen et al., 2016). Restriction of dietary protein levels in mice changes the content of small RNAs in mature sperm. More specifically, let-7 expression decreased and the expression level of tRFs & tiRNAs at the 5′ fragments of Glycine tRNA source increased. Therefore, tRFs & tiRNAs are also thought to play an endogenous reverse transcriptional role driving preimplantation transcriptome expression regulation in embryos (Sharma et al., 2016).

**FIGURE 1:**
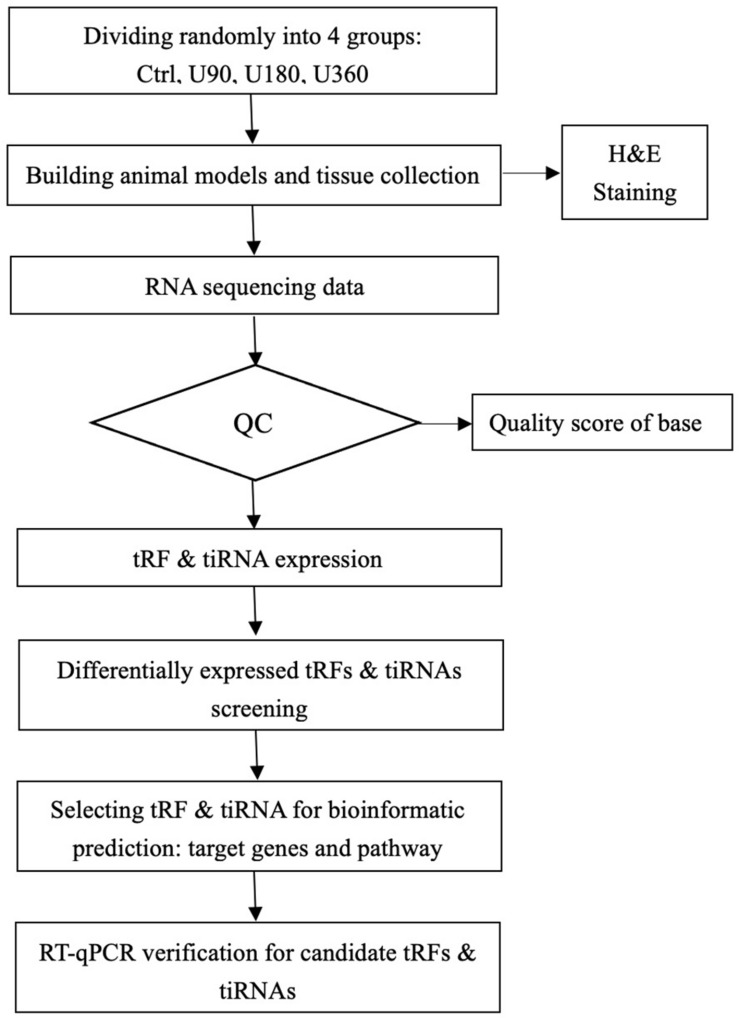
tRF & tiRNA-seq experiment workflow.

Presently, studies investigating tRFs & tiRNAs attracted increased attention, but no internal association between tRFs & tiRNAs and UV-induced skin damage has been analyzed. Therefore, the purpose of this study was to explore the spectrum of tRFs & tiRNAs and determine the mechanism behind tRFs & tiRNAs in UV damage. The research design for this study is presented in Figure 1.

## Materials and Methods

### Animals

A total of 12 mice (C57BL/6, adult males, aged 6–8 weeks and weighing about 18 ± 2 g, No. SCXK 2016-0002, March 29th, 2018) provided by the Central Animal Room of the Third Xiangya Hospital, Central South University were used in this study. Animals were kept in a humid environment with a constant temperature of 22–25°C and had free access to food and water. They also experienced a 12-h light/dark cycle. Experimental procedures were performed following laboratory requirements and were approved by the Bioethics Committee of Central South University.

Mice were randomly divided into 4 groups with 3 mice included in each group. The different experimental groups included the normal healthy control groups Ctrl (0 mJ/cm^2^), group U90 (90 mJ/cm^2^), group U180 (180 mJ/cm^2^), and group U360 (360 mJ/cm^2^).

Shaving: before the experiment, 1% pentobarbital sodium was used to perform preoperative anesthesia on C57BL/6 mice and 2 × 3 cm^2^ of back hair was removed using depilating cream and an electric pusher.

### Radiation Damage Model

Skin of mice in the experimental groups was irradiated using the SS-03B Ultraviolet Phototherapy Instrument (Shanghai Sigma High Technology Co., Ltd.). The irradiation dose used for the other groups was 90 mJ/cm^2^, 180 mJ/cm^2^, and 360 mJ/cm^2^ based on results from a preliminary experiment. Mice were placed on the work surface. The SS-03B Ultraviolet Phototherapy Instrument was preheated. The lamp tube height was adjusted 50 cm away from the workbench and mice were continuously irradiated for 8 days.

### Tissue Collection and Preservation

Changes and special conditions observed for mice before and after irradiation were recorded every day. After the 9th day, mice were sacrificed. Sterilized scissors were used to collect the back skin of experimental mice. Four pieces of skin tissue about 1 × 1 cm^2^ in size were collected from each mouse.

Removed skin tissue specimens were divided into two parts and stored. Half of the skin specimen was cut into pieces and frozen in liquid nitrogen for future use. The other half of the tissue specimen was placed in an EP tube and stored on dry ice for molecular detection. Each skin sample was marked with a corresponding label.

### Hematoxylin and Eosin (H&E) Staining

Fixed skin tissue samples were embedded in paraffin, sectioned at 4–5 μm and mounted on slides. Sections were deparaffinized, rehydrated in an ethanol gradient, and stained with H&E trichrome according to standard protocols using commercially available reagents.

### RNA Extraction and Quality Control

Total RNA using TRIzol reagent. Quality control (QC) using agarose gel electrophoresis was performed to detect the quantity and integrity of each RNA sample and then samples were quantified using a Nano drop ND-1000 instrument. Total RNA quantification and quality assurance was performed using a spectrophotometer.

### tRF & tiRNA Pretreatment

Total RNA samples were pretreated for removing tRF & tiRNA modifications which interfere with small RNA-seq library construction. Firstly, diacylated 3′-aminoacyl (charged) to 3′-OH for 3′ -adaptor ligation; secondly, removed 3′-cP (2′-cyclic phosphate) to 3′-OH for 3′ -adaptor ligation; thirdly, phosphorylated 5′-OH (hydroxyl group) to 5′-P for 5′-adaptor ligation; finally, demethylated m1A and m3C for more efficient reverse transcription.

### Library Preparation

The sequencing library was constructed by using the NEBNext^®^ Multiplex Small RNA Library Prep Set for Illumina^®^. Briefly, 3′-adapter and 5′-adapter ligated to RNA samples, then reverse transcription, PCR amplification and selected ∼134–160 bp PCR amplicons (corresponding to ∼14–40 nt small RNAs). The sequencing libraries were quantified using Agilent 2100 Bioanalyzer, and mixed with equal amounts based on quantification results and used for sequencing. The expression of tRF was annotated by referring to the tRNA sequence in the GtRNAdb database. Only fragments that can be compared with tRNA and pre-tRNA are defined as tRFs & tiRNAs. MiRNA and other small RNA fragments will not be compared with tRNA. Therefore, the influence of miRNA and other small RNA fragments can be excluded.

### Sequencing

The sequence reaction was performed on Illumina NextSeq 500 with NextSeq 500/550 V2 kit (#FC-404-2005, Illumina). Well mixed DNA libraries were denatured using 0.1 M NaOH for generating single strand DNA molecules and loading by NextSeq 500/550 V2 kit (#FC-404-2005, Illumina) according to the guidebook from manufacturer. A 50 sequencing was considered to be reasonable.

### Data Collection and Analysis

tRFs & tiRNAs levels were evaluated using sequencing counts and normalized as counts per million of total aligned reads (CPM). tRFs & tiRNAs differentially expressed were screened based on count values using the R package EdgeR (Robinson et al., 2010). Pie plots, Venn plots, Hierarchical clustering and Volcano plots were generated in R or using the perl environment for statistical computing and graphics.

### Target Genes Predictions for tRFs & tiRNAs

Some studies have used different algorithms to obtain possible seed sequences and targets for tRFs & tiRNAs referencing to miRNA target predictors (Kumar et al., 2014; Karaiskos and Grigoriev, 2016; Kim et al., 2017; Kuscu et al., 2018; Luo et al., 2018). Previous studies recommended using TargetScan and miRanda to predict tRFs & tiRNAs targets (Enright et al., 2003; Garcia et al., 2011; Li et al., 2020). A conclusion could only be drawn if both programs predicted genes to be targets mRNAs of the tRFs & tiRNAs.

### Bioinformatic Prediction

Metascape^1^ is a free, well-maintained, user-friendly gene-list analysis tool for gene annotation and analysis (Zhou et al., 2019). It is an automated meta-analysis tool to understand common and unique pathways within a group of orthogonal target-discovery studies. In this study, Metascape was used to conduct pathway and process enrichment analysis of tRF-Gly-CCC-019 target genes. For this, the Gene Ontology (GO) terms for biological process, cellular component, and molecular function categories, as well as Kyoto Encyclopedia of Genes and Genomes (KEGG) pathways, were enriched based on the Metascape online tool. Only terms with *P*-value < 0.01, minimum count of 3, and enrichment factor of >1.5 were considered as significant. The most statistically significant term within a cluster was chosen as the one representing the cluster. A subset of enriched terms was selected and rendered as a network plot to further determine the relationship among terms, where terms with similarity of >0.3 were connected by edges. Protein–protein interaction enrichment analysis was performed using the following databases: STRING, BioGrid, OmniPath, InWeb_IM. Further, Molecular Complex Detection (MCODE) algorithm was applied to identify densely connected network components. In this study, the “Express Analysis” module was used to further verify the enrichment of tRF-Gly-CCC-019 target genes.

### RT-qPCR

tRF-Gly-CCC-019, tRF-Trp-TCA-001, tRF-Val-AAC-012, and tRF-Val-CAC-018 were verified by RT-qPCR. Total RNAs of samples was extracted from sections using TRI Reagent (Sigma: T9424). RNAs concentration and purity were determined using NanoDrop^®^ ND-1000. RNAs integrity was tested by denatured agarosol gel electrophoresis. RNA pretreatment and cDNA synthesis were performed using rtStar^TM^ tRF & tiRNA Pretreatment Kit (Cat# AS-FS-005, Arraystar) and rtStar^TM^ First-Strand cDNA Synthesis Kit (Cat# AS-FS-003, Arraystar), respectively. The 2× PCR master mix (Arraystar:AS-MR-006-5) was used for the PCR through ViiA 7 Real-time PCR System (Applied Biosystems). The reactions were incubated at 95°C for 10 min, followed by 40 cycles at 95°C for 10 s, 60°C for 60 s (fluorescent signals were measured). The results were normalized to the reference of U6RNA and calculated.

### Statistical Analysis

GraphPad prism software was used for statistical analysis in RT-qPCR. Results are presented as mean ± standard deviation (SD). tRFs & tiRNAs expression belongs to discrete distribution. Significant differences between the groups were compared using the negative binomial distribution. tRFs & tiRNAs differentially expressed were screened based on count values using the R package EdgeR (Robinson et al., 2010). The significance level was set at *P* ≤0.05.

### Database and Accession Numbers

The raw data of the tRFs & tiRNAs-seq in our study was submitted in Gene Expression Omnibus (GEO), which were assigned GEO accession numbers GSE162641. Our series entry (GSE162641^2^) provides access to all of our data.

## Results

### Mouse Skin Injury Induced by UVB Irradiation

**FIGURE 2:**
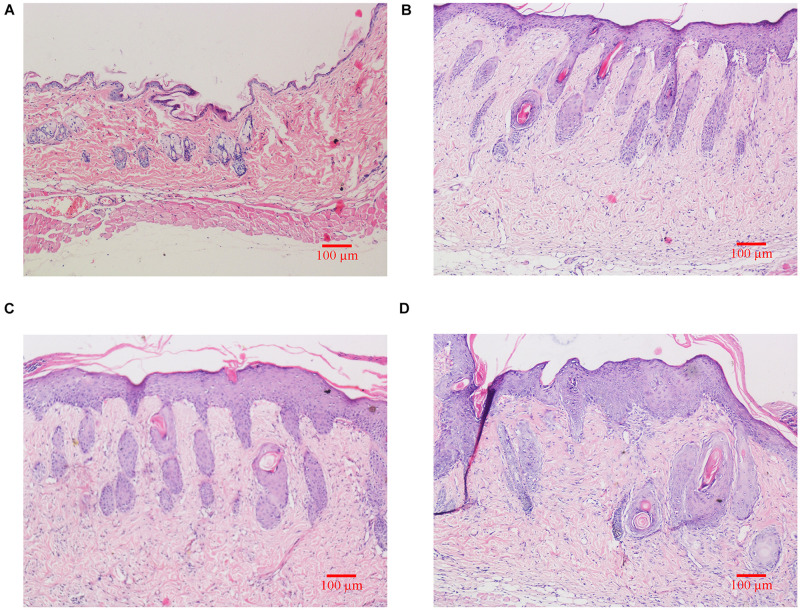
Histological changes of dorsal skin in mice exposed to different doses of UVB irradiation. These show the magnification is 10 times under an optical microscope. **(A)** Control group; **(B)** The experimental group received radiation dose of 90 mJ/cm^2^; **(C)** The experimental group received radiation dose of 180 mJ/cm^2^; **(D)** The experimental group received radiation dose of 360 mJ/cm^2^.

As shown in Figure 2, H&E staining results revealed that skin structure of control group Ctrl was intact (see Figure 2A). The skin epidermis was evenly distributed without obvious thickening and the dermal papilla layer was clear. The basement membrane was not degraded no inflammatory cells were observed. In groups exposed to UVB irradiation (see Figures 2B–D), the skin structure was incomplete. The cuticle was detached and the epidermal layer was significantly thickened, which was significantly different from the control group. The epidermal basement membrane was degraded. Inflammatory cells infiltrated into the epidermis, and the dermal tissue was disordered.

### Altered Expression Profiles of tRFs & tiRNAs

**FIGURE 3:**
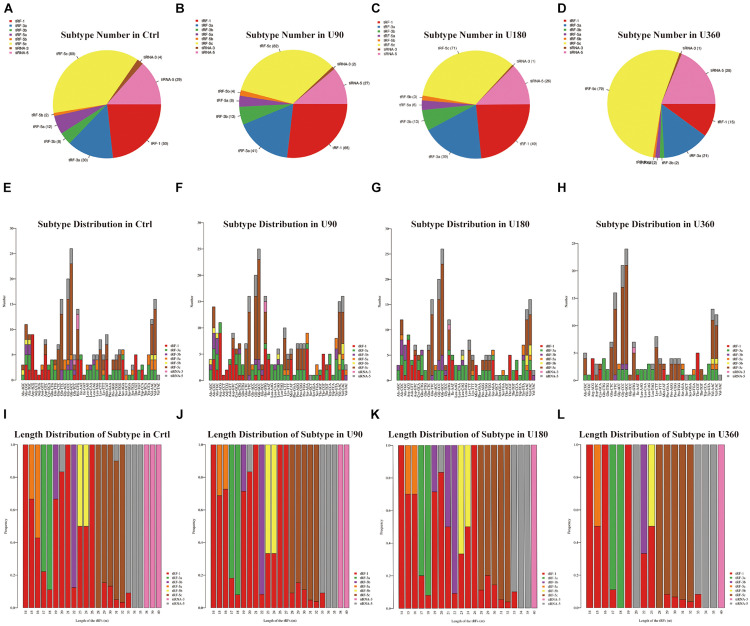
The analysis of subtypes tRF & tiRNA. **(A–D)** Pie chart of the distribution of subtypes tRF & tiRNA. The values in bracket are represented the number of subtypes tRF & tiRNA. The color represents the subtypes tRF & tiRNA. **(E–H)** The number of subtypes tRF & tiRNA against tRNA isodecoders. The *X* axis represents tRNA isodecoders and the *Y* axis show the number of all subtypes tRF & tiRNA. **(I–L)** The frequency of subtype against length of the tRF & tiRNA. The *X* axis represents length of tRF & tiRNA and the *Y* axis show the frequency of the subtypes against length of tRF & tiRNA.

tRF & tiRNA-Seq was used to identify tRF & tiRNA expression levels in these groups. The first step was to evaluate and test the sequencing quality and draw a QC graph for each sample. The Q is related to the logarithm of the base, the quality score plot of each sample was plotted (see Supplementary Figure 1 and Supplementary Table 1). call error probability (P): *Q* = −10log_10_(P). For instance, Q30 implies an incorrect base call probability of 0.001 or 99.9% base call accuracy. According to the results of Figure 3 and Table 1, sample quality was qualified.

**TABLE 1:** The candidate tRF & tiRNA for bioinformatics.

**Name**	**Groups**	**Type**	**Length**	**Fold change**	***P*-value**	***Q*-value**	**Regulation**
tRF-Pro-AGG-012	B:A	tRF-5c	31	4.781440594	0.003103144	0.038175315	Up
	C:A	tRF-5c	31	4.05575894	0.005132839	0.053480873	Up
	D:A	tRF-5c	31	7.420759879	8.98333E-08	7.25404E-06	Up
tRF-Val-AAC-012	B:A	tRF-5c	32	3.016918321	0.04193729	0.191943749	Up
	C:A	tRF-5c	32	5.3389584	0.002805139	0.040582801	Up
	D:A	tRF-5c	32	6.583554544	0.000271897	0.002661296	Up
tRF-Val-AAC-031	B:A	tRF-5c	32	8.402013832	2.71448E-05	0.003230235	Up
	C:A	tRF-5c	32	9.802656488	1.05991E-05	0.003423517	Up
	D:A	tRF-5c	32	9.370313038	3.21793E-06	0.000124659	Up
tRF-Val-CAC-018	B:A	tRF-5c	32	5.129605552	0.001975966	0.033591418	Up
	C:A	tRF-5c	32	7.603784623	0.000229387	0.012348652	Up
	D:A	tRF-5c	32	6.590396309	0.000186961	0.002208657	Up
tiRNA-Ser-GCT-001	B:A	tiRNA-5	20	0.048863505	0.000424766	0.014018123	Down
	C:A	tiRNA-5	20	0.089925461	0.001668834	0.036500014	Down
	D:A	tiRNA-5	20	0.08419616	0.001730948	0.009476206	Down
tRF-Ala-TGC-001	B:A	tRF-1	20	0.285374208	0.00924715	0.072428766	Down
	C:A	tRF-1	20	0.245661274	0.003809353	0.048234951	Down
	D:A	tRF-1	20	0.129291736	5.32853E-05	0.000819578	Down
tRF-Ala-TGC-002	B:A	tRF-1	21	0.378947046	0.017991463	0.110740554	Down
	C:A	tRF-1	21	0.249866758	0.002035664	0.03867762	Down
	D:A	tRF-1	21	0.249429607	0.00166444	0.009431824	Down
tRF-Arg-CCT-002	B:A	tRF-1	15	0.420313273	0.029433192	0.147995064	Down
	C:A	tRF-1	15	0.213410181	0.000454298	0.016304266	Down
	D:A	tRF-1	15	0.038656323	3.25305E-10	5.25368E-08	Down
tRF-Gly-CCC-019	B:A	tRF-5a	16	0.175976904	0.002099658	0.034071729	Down
	C:A	tRF-5a	16	0.201466914	0.00275907	0.040582801	Down
	D:A	tRF-5a	16	0.235544725	0.006476565	0.026818485	Down
tRF-Trp-TCA-001	B:A	tRF-3a	17	0.11768156	0.003975342	0.044329859	Down
	C:A	tRF-3a	17	0.087855086	0.000178682	0.012348652	Down
	D:A	tRF-3a	17	0.066019654	5.26313E-05	0.000819578	Down

A Venn diagram showed commonly expressed and specifically expressed tRFs & tiRNAs. In Supplementary Figure 2D, the Venn diagram revealed known tRFs from tRFdb (Kumar et al., 2014, 2015) and the detected tRFs & tiRNAs in this experiment. There were 57 tRFs & tiRNAs observed in both two groups which represented common tRFs & tiRNAs. Additionally, 269 specific tRFs & tiRNAs were detected that were not previously known.

In Figure 3, a pie chart was used to describe each subtype of tRF & tiRNA. The pie chart showed the distribution of each subtype where the CPM of the sample or the average CPM of the group was not less than 20. In these tRFs & tiRNAs, the expression levels of tRF-3a increased, whereas tRF-5a and tiRNA-3 levels decreased compared to the control (Ctrl). Furthermore, the number of tRFs & tiRNAs subtypes were counted against tRNA isodecoders. The stacked bar chart (see Figures 3E–H) represents different tRNA isodecoders on top of one another. The height of the resulting bar showed the combined result of tRNA isodecoders. The frequency of tRFs & tiRNAs subtypes can be calculated against the length of the sequence (see Figures 3I–L). The height of the resulting bar showed the combined results of tRFs & tiRNAs length.

Hierarchical clustering was arranged to analyze tRF & tiRNA expression data. Each row represented one tRF & tiRNA and all that were selected were categorized into no more than 10 clusters based on K-means clustering. Each column represents one sample (see Supplementary Figures 3A–C). The following scatter plots in Supplementary Figures 3D–F were a visualization method for assessing the tRF & tiRNA expression variation. Also, Volcano plots provide a quick visual identification of the tRFs & tiRNAs displaying large magnitude changes that were statistically significant (see Supplementary Figures 3G–I). Next, the selection of tRF & tiRNA criteria included a higher FC, lower *q*-value and higher CMP. After a unified summary of the original data, 10 differentially expressed tRFs & tiRNAs were screened, among which 4 tRFs & tiRNAs were significantly up-regulated and 6 tRFs & tiRNAs were significantly down-regulated (see Table 1).

### Bioinformatic Prediction

The 10 differentially expressed tRFs & tiRNAs included tRF-Arg-CCT-002, tRF-Trp-TCA-001, tiRNA-Ser-GCT-001, tRF-Gly-CCC-019, tRF-Ala-TGC-001, tRF-Ala-TGC-002, tRF-Val-AAC-012, tRF-Pro-AGG-012, tRF-Val-CAC-018, and tRF-Val-AAC-031. Target genes were predicted for these 10 tRFs & tiRNAs. To improve prediction reliability, targetScan (Garcia et al., 2011) and miRanda (Enright et al., 2003) were used to predict the target genes of differentially expressed tRFs & tiRNAs, respectively.

**FIGURE 4:**
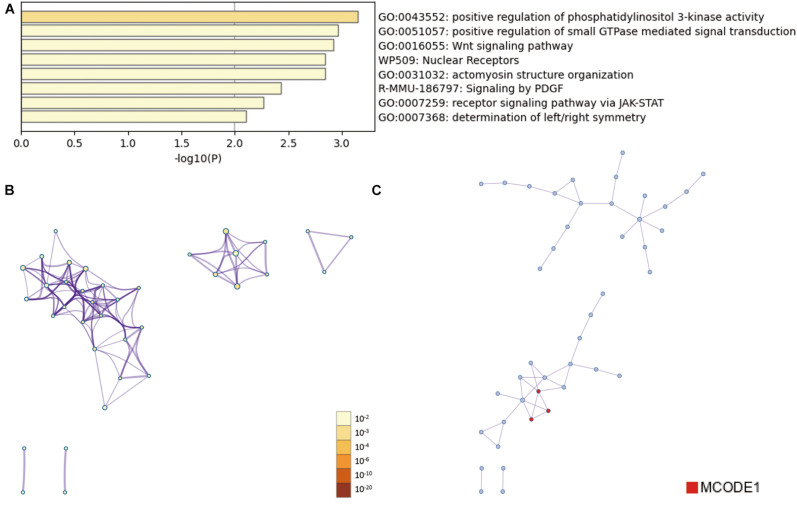
Bioinformatic Prediction. **(A)** Heatmap enriched terms colored by *p*-values. **(B)** Network of enriched terms colored by *p*-value, where terms containing more genes tend to have a more significant *p*-value. **(C)** Protein-protein interaction (PPI) network.

Then, the functions enrichment analysis of tRF-Gly-CCC-019 target genes were obtained from Metascape. As presented in Figure 4A, the functions of differentially expressed tRF-Gly-CCC-019 target genes were mainly enriched in Wnt signaling pathway, positive regulation of phosphatidylinositol 3-kinase activity, signaling by PDGF, receptor signaling pathway via JAK-STAT, actomyosin structure organization, positive regulation of small GTPase mediated signal transduction, determination of left/right symmetry and Nuclear Receptors. Among these pathways, the Wnt signaling pathway and JAK-STAT signaling pathway were found to be related to injury induced by ultraviolet irradiation. In addition, to better understand the relationship between tRF-Gly-CCC-019 and skin injury, we performed a Metascape protein–protein interaction enrichment analysis. The protein–protein interaction network and MCODE components identified in the gene lists are shown in Figures 4B,C. After pathway and process enrichment analysis was independently applied to each MCODE component, the results showed that biological function was mainly related to regulation of ERK1 and ERK2 cascade (see Table 2). The target gene Rac1 predicted by tRF-Trp-TCA-001 and tRF-Gly-CCC-019 was enriched in the WNT signaling pathway. Therefore, it was speculated that tRF-Gly-CCC-019 play important roles in acute skin injury induced by UVB radiation by regulating the ras-related C3 botulinum toxin substrate 1 (Rac1) gene involved in the WNT signaling pathway.

**TABLE 2:** MCODE.

**MCODE**	**GO**	**Description**	**Log10(P)**
MCODE_1	GO:0070374	positive regulation of ERK1 and ERK2 cascade	−5.9
MCODE_1	GO:0070372	regulation of ERK1 and ERK2 cascade	−5.4
MCODE_1	GO:0070371	ERK1 and ERK2 cascade	−5.4

### RT-qPCR Verification

**FIGURE 5:**
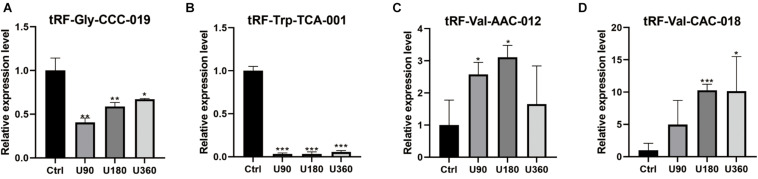
Validation of the four selected tsRNAs using RT-qPCR verification for four tRFs & tiRNAs. Compared with the control group A, **(A,B)** tRF-Gly-CCC-019 and tRF-Trp-TCA-001 were down-regulated; **(C,D)** tRF-Val-AAC-012 and tRF-Val-CAC-018 were up-regulated. The data were normalized using the mean ± standard error of the mean (SEM). *P ≤0.05, **P ≤0.01, ***P ≤0.001.

tRF-Gly-CCC-019, tRF-Trp-TCA-001, tRF-Val-AAC-012, and tRF-Val-CAC-018 expressions were verified by RT-PCR (see Figure 5). *T*-test was used for statistical analysis of the relative expression levels, and the results showed that *P* ≤0.05 was statistically significant. Compared with the control group, tRF-Val-AAC-012 and tRF-Val-CAC-018 were significantly up-regulated; tRF-Gly-CCC-019 and tRF-Trp-TCA-001 were significantly down-regulated. Inappropriate preservation and usage of individual specimens resulted in intra-group differences, but it did not affect the overall trends. In particular, the downregulation trends of tRF-Gly-CCC-019 and tRF-Trp-TCA-001 were significant and all of them were statistically significant. Then we focused on them and predicted their target gene and signaling pathway. Primers were designed using software (Primer 5.0) whose sequences were shown in the Table 3.

**TABLE 3:** Sequences of primers for RT-qPCR.

**Gene_ID**	**Primer sequences**	**Ta Opt (°C)**	**Product size (bp)**
U6	F:5′ GCTTCGGCAGCACATATACTAAAAT 3′ R:5′ CGCTTCACGAATTTGCGTGTCAT 3′	60	89
tRF-Gly- CCC-019	F:5′ ACAGTCCGACGATCGCATTG 3′ R:5′ GTGCTCTTCCGATCTACTGAACCA 3′	60	45
tRF-Trp- TCA-001	F:5′ GTTCTACAGTCCGACGATCAAGT 3′ R:5′ TTCCGATCTTGGCAGAAGTTA 3′	60	45
tRF-Val- AAC-012	F:5′ GATCGTTTCCGTAGTGTAGTGGTCA 3′ R:5′ CTTCCGATCTGGCGAGCGT 3′	60	46
tRF-Val- CAC-018	F:5′ TACAGTCCGACGATCGTTTCC 3′ R:5′ CGATCTGGCGAGCGTGATA 3′	60	53

## Discussion

Here, we revealed the tRF & tiRNA expression profiles of skin damaged by UVB using RNA sequencing. An animal model was generated to simulate skin injury caused by UVB radiation and RNA sequencing was performed on skin samples to screen 10 tRFs & tiRNAs that were differentially expressed. We found that tRF-Trp-TCA-001 and tRF-Gly-CCC-019 regulated the Rac1 gene through the WNT signaling pathway, which likely revealed the mechanism behind UVB radiation damage, provided a diagnostic basis for ultraviolet radiation damage and provided auxiliary targets for treatment.

tiRNAs are 5′- and 3′ -tRNA halves produced by specific cleavage of the anticodon loops of mature tRNAs with angiogenin (ANG) under various stress conditions. tRFs are derived from mature tRNAs or pre-tRNAs. According to their corresponding positions on tRNAs, tRFs can further be divided into four types including tRF-5, tRF-3, tRF-1, and tRF-2 (Kumar et al., 2015; Yu et al., 2021). tRF-5 corresponds to the 5′ end of mature tRNAs and cutting occurs in the D-loop. tRF-3 corresponds to the 3′end of mature tRNAs and contains CCA and cutting occurs in the T-loop (Kirchner and Ignatova, 2015). tRF-1 is derived from the 3′ tail sequence of the precursor tRNA, which contains a poly U sequence at the 3′ end. tRF-2, not belonging to tRF-5, tRF-3, or tRF-1, comes primarily from the intermediate region of mature tRNAs (Goodarzi et al., 2015; Wang et al., 2020; Yu et al., 2021). tRFs & tiRNAs play biological roles through a variety of mechanisms, including interacting with proteins or mRNAs, regulating gene expression, controlling cell cycle, regulating chromatin and epigenetic modifications (Guzzi et al., 2018; Xie et al., 2020; Li et al., 2021).

Studies have shown that when skin keratinocytes are subjected to stress, the production of additional tRFs & tiRNAs increases and play a role in translation (Pliatsika et al., 2018; Grafanaki et al., 2019). Both tRFs & tiRNAs have also been identified as a novel biomarker for the treatment of skin melanoma (Zheng et al., 2016). Other studies have shown that tsRNAs are potential therapeutic targets for caloric restriction pretreatment to improve myocardial ischemic injury (Liu et al., 2020). Moreover, tsRNA is a potential therapeutic target for Buyang-Huanwu-Decoction (BYHWD) in the treatment of intracerebral hemorrhage (ICH), which provides new insights into the mechanism by which BYHWD promotes neural function recovery after ICH (Li et al., 2019). Gu et al. (2020) revealed peripheral blood tsRNAs as novel biomarkers in lung cancer. However, the relationship between tRFs & tiRNAs as well as human skin injury has not been reported so it is necessary to conduct in-depth research on the expression levels and roles of tRFs & tiRNAs in skin after injury.

tRNA-derived small RNAs (tsRNAs) are fragments derived from tRNAs, that have been of interest to researchers over the years. Therefore, RNA sequencing technology was used to look for differentially expressed tRFs & tiRNAs in UV-damaged skin tissues and normal skin tissues and to explore the mechanisms behind UV-damaged skin tissue. After RNA sequencing of tRFs & tiRNAs, screening was performed according to higher FC, lower *P*-value, lower *q*-value and higher CMP, and four up-regulated tRFs & tiRNAs were selected including tRF-Val-AAC-012, tRF-Pro-AGG-012, tRF-Val-CAC-018, and tRF-Val-AAC-031. Six tRFs & tiRNAs were significantly down-regulated, including tRF-Arg-CCT-002, tRF-Trp-TCA-001, tiRNA-Ser-GCT-001, tRF-Gly-CCC-019, tRF-Ala-TGC-001 and tRF-Ala-TGC-002. Next, tRF & tiRNA target genes were predicted based on screened tRFs & tiRNAs. Metascape analysis results were performed based on predicted results of target genes. Compared with the control group, target genes by tRF-Gly-CCC-019 were mainly concentrated in Wnt signaling pathway, positive regulation of phosphatidylinositol 3-kinase activity, signaling by PDGF, receptor signaling pathway via JAK-STAT and so on.

Besides tRF-Gly-CCC-019, there are also target genes predicted by tRF-Trp-TCA-001 that act on Wnt signaling pathway. All of these confirmed that Wnt signaling pathway may plays a key role in UV induced skin injury. Up regulated tRF-Val-AAC-012, tRF-Pro-AGG-012, and tRF-Val-CAC-018 target genes were enriched in RTKs (receptor tyrosine kinases) signaling pathway. RTKs signaling pathway is closely related to inflammation, tumor development and deterioration. All these suggest that we can broaden the application scope of tRF-Val-AAC-012, tRF-Pro-AGG-012, and tRF-Val-CAC-018. In addition to the field of injury, it may become an indicator of inflammation and even a tumor marker in clinic. Whereas, the clinical application of tRFs & tiRNAs needs further evaluation. tRFs & tiRNAs have been shown to be differentially expressed in various diseases, such as trauma, cancer, metabolic diseases and so on. All of these emphasize their potential value in clinical application. In the process of acute tissue and organ injury, the level of tRFs & tiRNAs in circulatory system increased. This will make tRFs & tiRNAs more sensitive than other known tissue damage markers. According to the results of this experiment, it is expected that tRFs & tiRNAs can be used as a new marker to identify tissue damage in the future. However, only tRFs & tiRNAs in skin tissue have been detected. It is hoped that more tissues such as organs can be detected in future research. In order to develop more appropriate detection methods and establish more comprehensive clinical indicators, provide the basis for clinical diagnosis and treatment.

Metascape showed that tRF-Gly-CCC-019 target genes were significantly enriched in the WNT signaling pathways. Wu et al. (2019) indicated that treatment with NF157 prevented UV-B irradiation-induced destruction of the Wnt/β-catenin signaling transduction pathway by increasing the expression of Wnt1, Wnt3a, c-Myc, and cyclin D1. Shen et al. (2019) reported the occurrence of photoaging on the skin, which was characterized by UV-damage caused by matrix degradation and inflammatory changes involving the WNT pathway. These studies indicate that the WNT pathway is closely related to UV-damage, which is consistent with our predictions. The target gene Rac1 predicted by tRF-Trp-TCA-001 and tRF-Gly-CCC-019 was enriched in the WNT signaling pathway. Rac1 regulates the DNA injury response and protects from UV-induced keratinocyte apoptosis and skin carcinogenesis (Deshmukh et al., 2017). UV signaling and an activation mutation in RAC1 was identified in 9.2% of melanoma patients exposed to sunlight. These results are consistent with the results presented in this study (Krauthammer et al., 2012). We speculated that tRF-Gly-CCC-019 regulate the Rac1 gene through the WNT signaling pathway, thus playing a role in skin damage caused by UV radiation.

Here, we compared three UV treatment groups with the control group and constructed plots to reveal the results. In future studies, it would be best to compare between the three UV treated groups. Despite these findings, our study still faced certain limitations. First of all, tRNAs have many different chemical modifications, some that may hinder library preparation and sequencing, ultimately leading to bias. Secondly, our sequencing is aimed at mouse skin, which cannot be completely analogous to human genes. Hence, in the future we plan to perform more in-depth research to improve our studies.

Given that the relationship between tRFs & tiRNAs and human skin injury has not been previously reported, we know very little regarding the function these tRNA-derived RNAs exert. Nonetheless, the expression of these tRFs & tiRNAs significantly different in skin injury induced by UV, which suggests that tRFs & tiRNAs may contain important, yet to be determined roles in UV-damage. Thus, our research may provide further insight into the pathogenesis of diseases and may contribute to the development of new therapies.

## Data Availability Statement

The datasets presented in this study can be found in online repositories. The names of the repository/repositories and accession number(s) can be found below: Gene Expression Omnibus (GEO) database under accession number GSE162641 (https://www.ncbi.nlm.nih.gov/geo/query/acc.cgi?acc=GSE162641). All submitted data can be found in the Supplementary Material including raw data and original data.

## Ethics Statement

The animal study was reviewed and approved by the Bioethics Committee of Central South University.

## Author Contributions

YF, YL, and XL performed the research. JZ and KC designed the research study. ZX, CP, XC, and YZ contributed essential reagents or tools. YY, YS, and ZL helped to analyze the data. YF wrote the manuscript. JZ revised the manuscript. All authors read and approved the final manuscript.

## Conflict of Interest

The authors declare that the research was conducted in the absence of any commercial or financial relationships that could be construed as a potential conflict of interest.

## Publisher’s Note

All claims expressed in this article are solely those of the authors and do not necessarily represent those of their affiliated organizations, or those of the publisher, the editors and the reviewers. Any product that may be evaluated in this article, or claim that may be made by its manufacturer, is not guaranteed or endorsed by the publisher.

## Footnotes

1
http://metascape.org

2
https://www.ncbi.nlm.nih.gov/geo/query/acc.cgi?acc=GSE162641
