# Supplementary material for: Differential Expression Profiles and Function Predictions for tRFs & tiRNAs in Skin Injury Induced by Ultraviolet Irradiation
Source: Front Cell Dev Biol. 2021 Aug 10;9:707572. doi: 10.3389/fcell.2021.707572 (PMC8383935; doi:10.3389/fcell.2021.707572)
Supplement: Supplementary file 3 [file Data_Sheet_1.docx]

Supplementary Material

# Supplementary Data

**Supplementary Figure 1.** tRF & tiRNA-seq quality score plot. The position in the read is plotted on the X-axis and the Q value is plotted on the Y-axis. The red line is the median Q score, and the blue line is the mean Q score. The boxplot represents the inter-quartile range, while the whiskers represent the 10% and 90% points. Q score above 30 (>99.9% correct) is considered high quality data. (A)-(L) respectively represents the quality score plot of different samples.

**Supplementary Figure 2.** Venn diagram. (A)-(C) These diagram shows the number of tRFs & tiRNAs which expressed in both of two groups and indicated the number of specific expressed tRFs & tiRNAs. (D) This diagram shows the number of tRFs & tiRNAs detected and collected in the tRFdb.

**Supplementary Figure 3.** Differentially expressed tRF & tiRNA screening. (A)-(C) The hierarchical clustering heatmap for tRF & tiRNA. The color scale is show below: blue represents an expression level below the mean, and red represents an expression lever above the mean. (D)-(F) These respectively shows the scatter plot of U90 vs Ctrl, U180 vs Ctrl and U360 vs Ctrl. (G)-(I) The volcano plot. Red circles indicate statistically up-regulated expression, green circles indicate down-regulated, and grey circles indicate non-differentially expressed tRF & tiRNA.

# Supplementary Tables

Table 1. tRF & tiRNA-seq quality score.

| Sample | Total Base | Base Q30 | Base Q30 (%) |
| --- | --- | --- | --- |
| A1 | 786510219 | 702167780 | 89.28 |
| A2 | 424889501 | 470392668 | 89.62 |
| A3 | 405023487 | 357701492 | 88.32 |
| B1 | 311354643 | 279215172 | 89.68 |
| B2 | 417968052 | 383393493 | 91.73 |
| B3 | 408903363 | 379315329 | 92.76 |
| C1 | 403061364 | 373430867 | 92.65 |
| C2 | 450918795 | 417050906 | 92.49 |
| C3 | 474580245 | 439899327 | 92.69 |
| D1 | 497716293 | 459828608 | 92.39 |
| D2 | 554615820 | 514129493 | 92.70 |
| D3 | 470435679 | 435122166 | 92.49 |

## Supplementary Figures


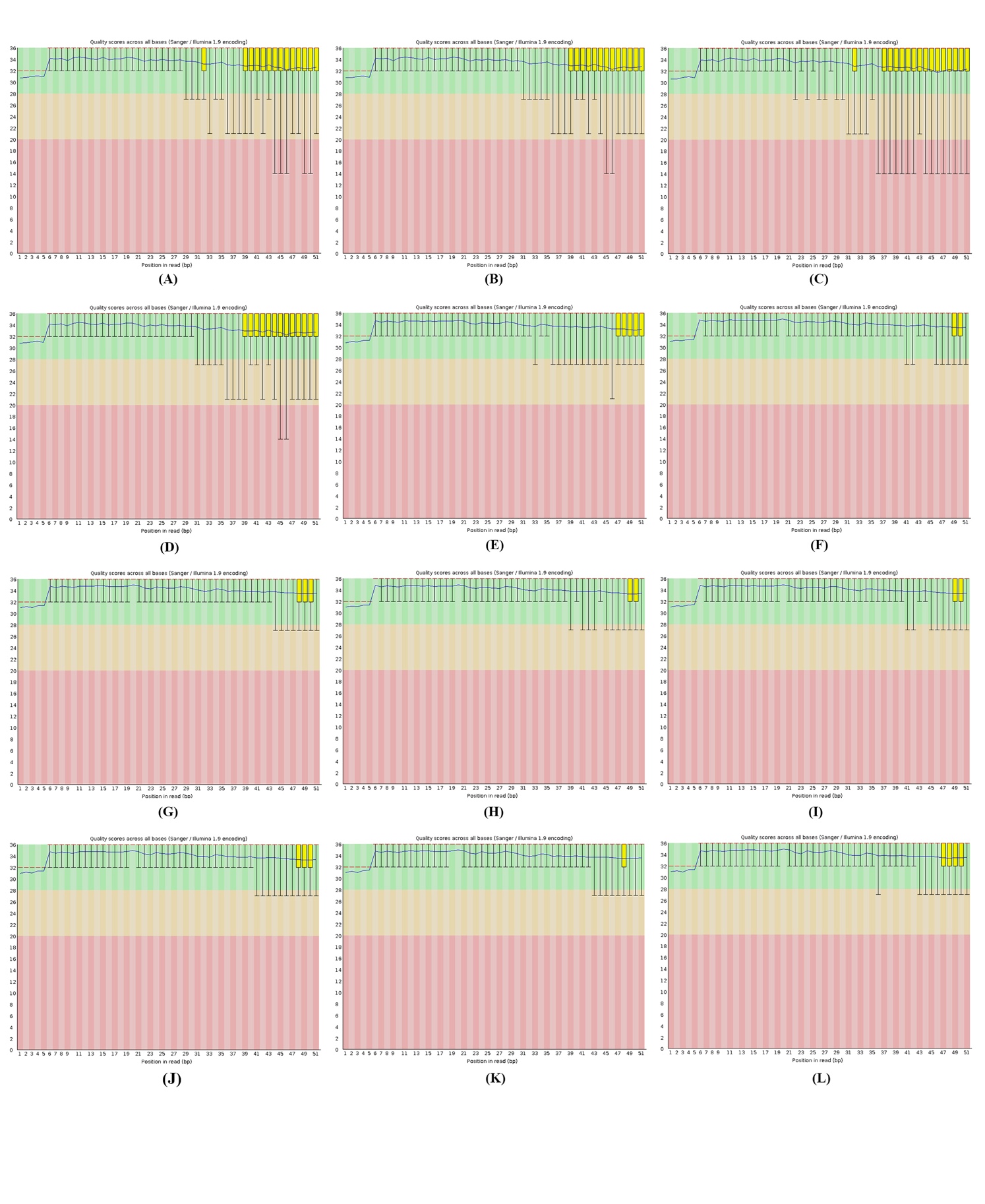


**Supplementary Figure 1.** tRF & tiRNA-seq quality score plot. The position in the read is plotted on the X-axis and the Q value is plotted on the Y-axis. The red line is the median Q score, and the blue line is the mean Q score. The boxplot represents the inter-quartile range, while the whiskers represent the 10% and 90% points. Q score above 30 (>99.9% correct) is considered high quality data. (A)-(L) respectively represents the quality score plot of different samples.


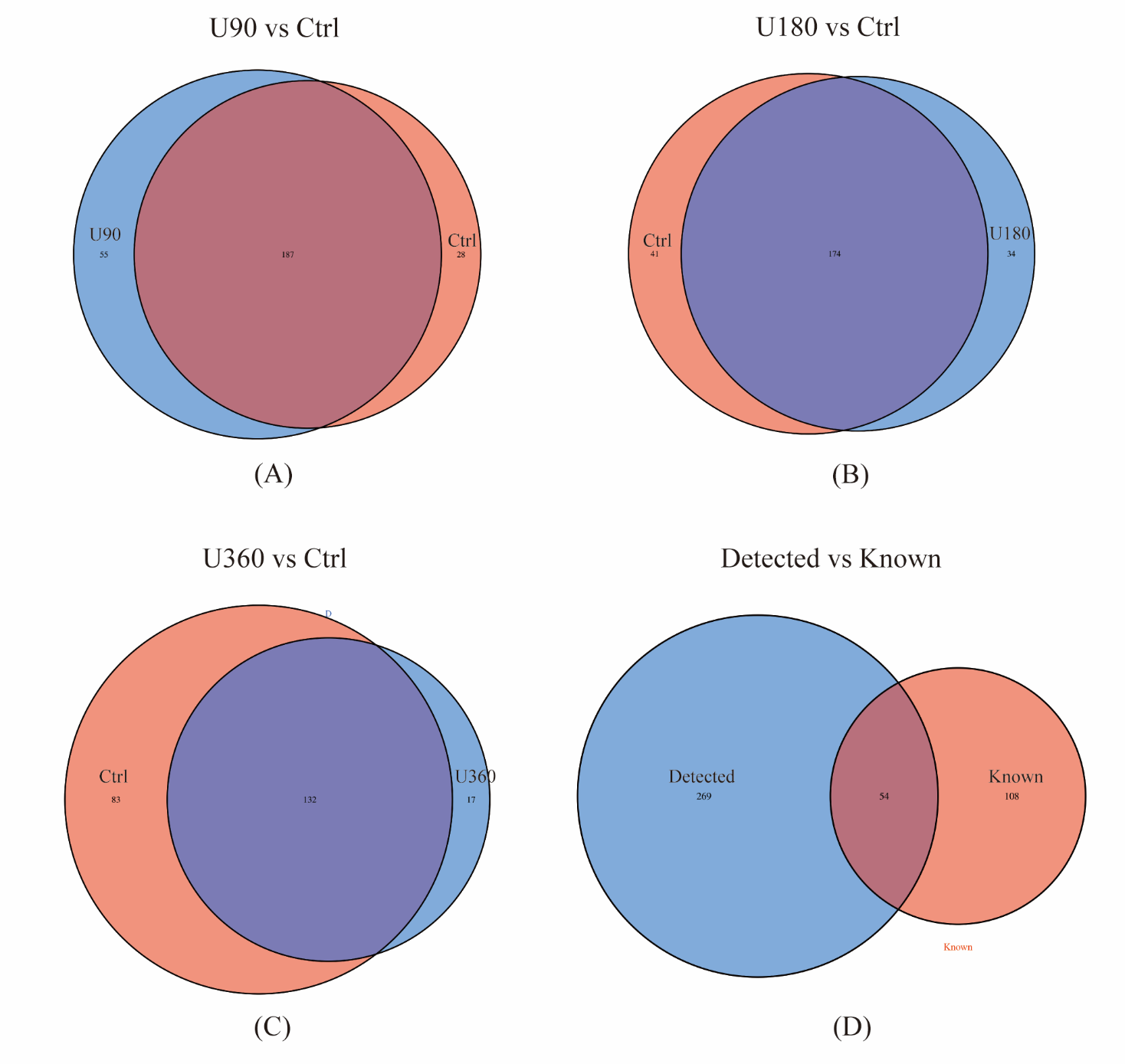


**Supplementary Figure 2.** Venn diagram. (A)-(C) These diagram shows the number of tRFs & tiRNAs which expressed in both of two groups and indicated the number of specific expressed tRFs & tiRNAs. (D) This diagram shows the number of tRFs & tiRNAs detected and collected in the tRFdb.


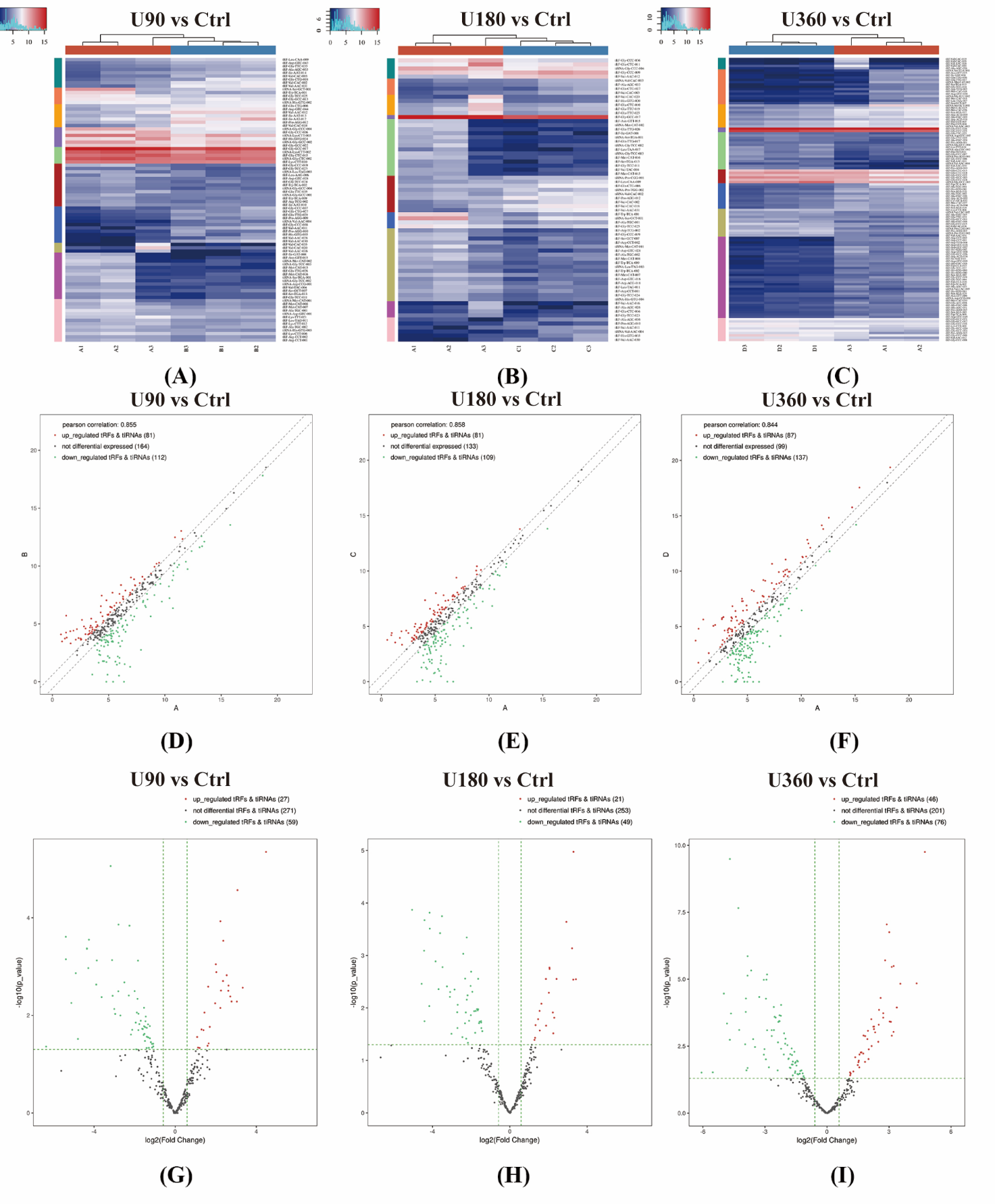


**Supplementary Figure 3.** Differentially expressed tRF & tiRNA screening. (A)-(C) The hierarchical clustering heatmap for tRF & tiRNA. The color scale is show below: blue represents an expression level below the mean, and red represents an expression lever above the mean. (D)-(F) These respectively shows the scatter plot of U90 vs Ctrl, U180 vs Ctrl and U360 vs Ctrl. (G)-(I) The volcano plot. Red circles indicate statistically up-regulated expression, green circles indicate down-regulated, and grey circles indicate non-differentially expressed tRF & tiRNA.
