# Supplementary figures and images for: Differential Expression Profiles and Function Predictions for tRFs & tiRNAs in Skin Injury Induced by Ultraviolet Irradiation
Source: Front Cell Dev Biol. 2021 Aug 10;9:707572. doi: 10.3389/fcell.2021.707572 (PMC8383935; doi:10.3389/fcell.2021.707572)

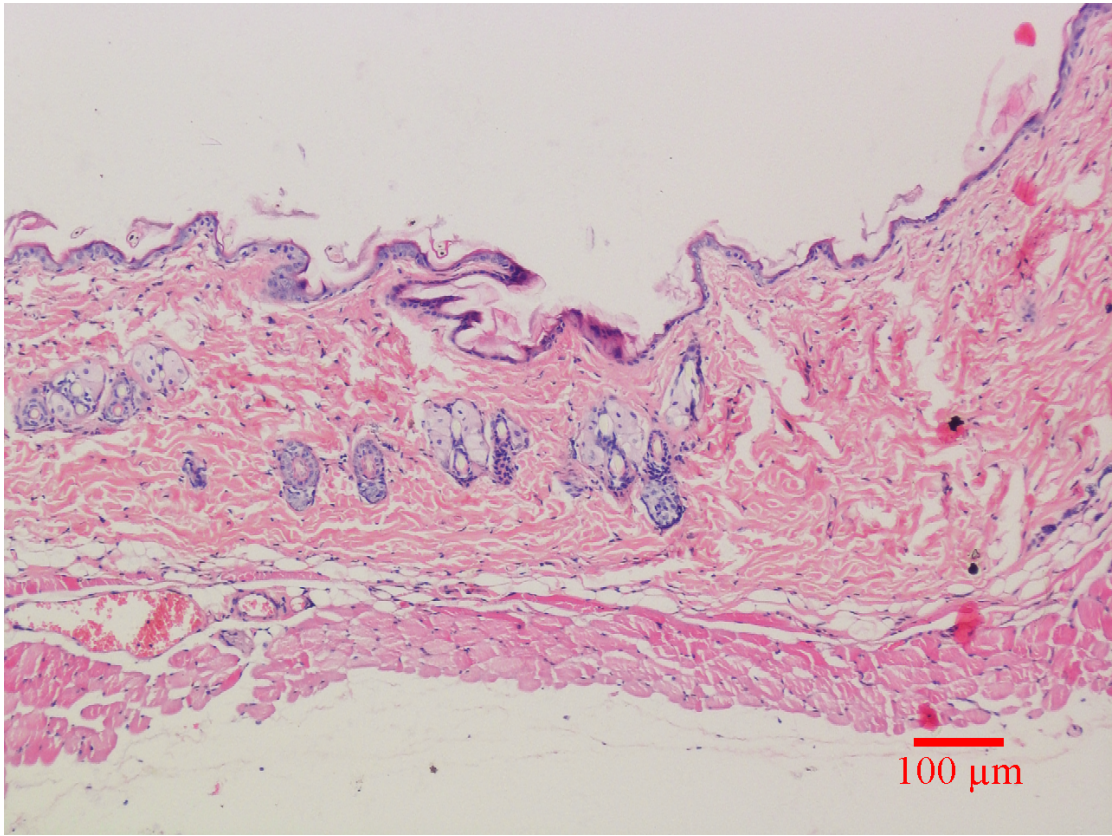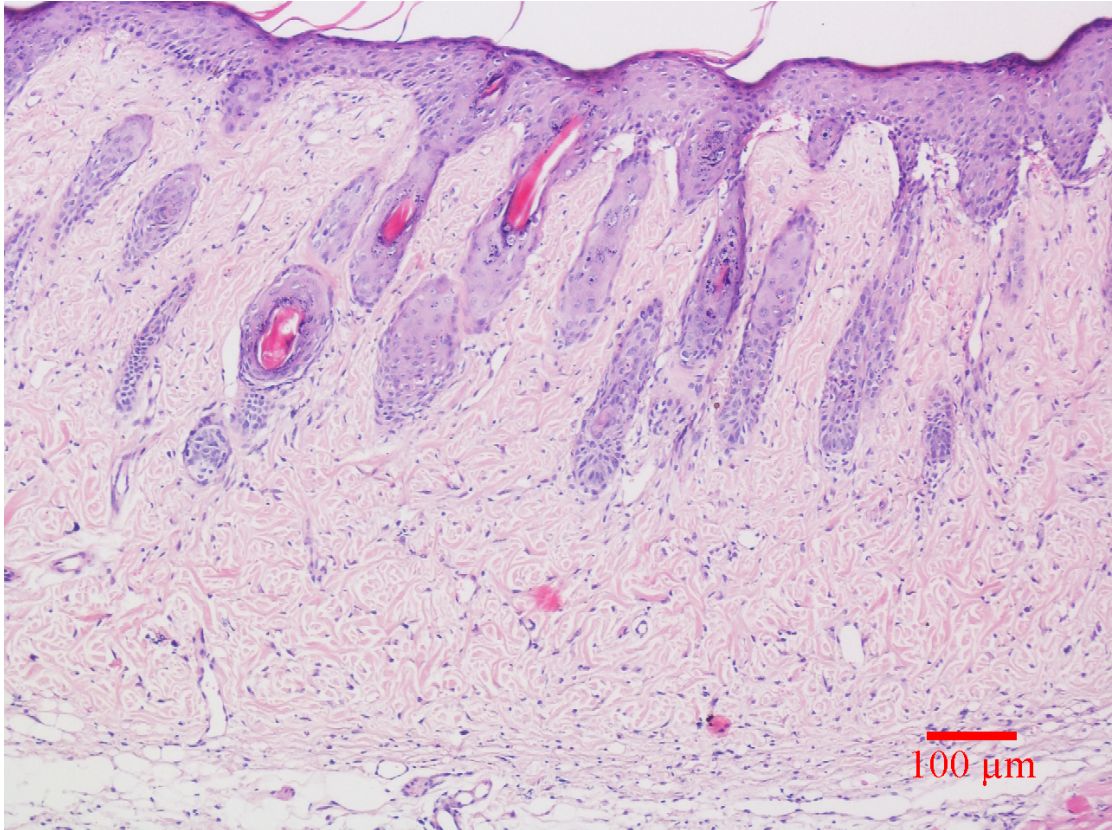

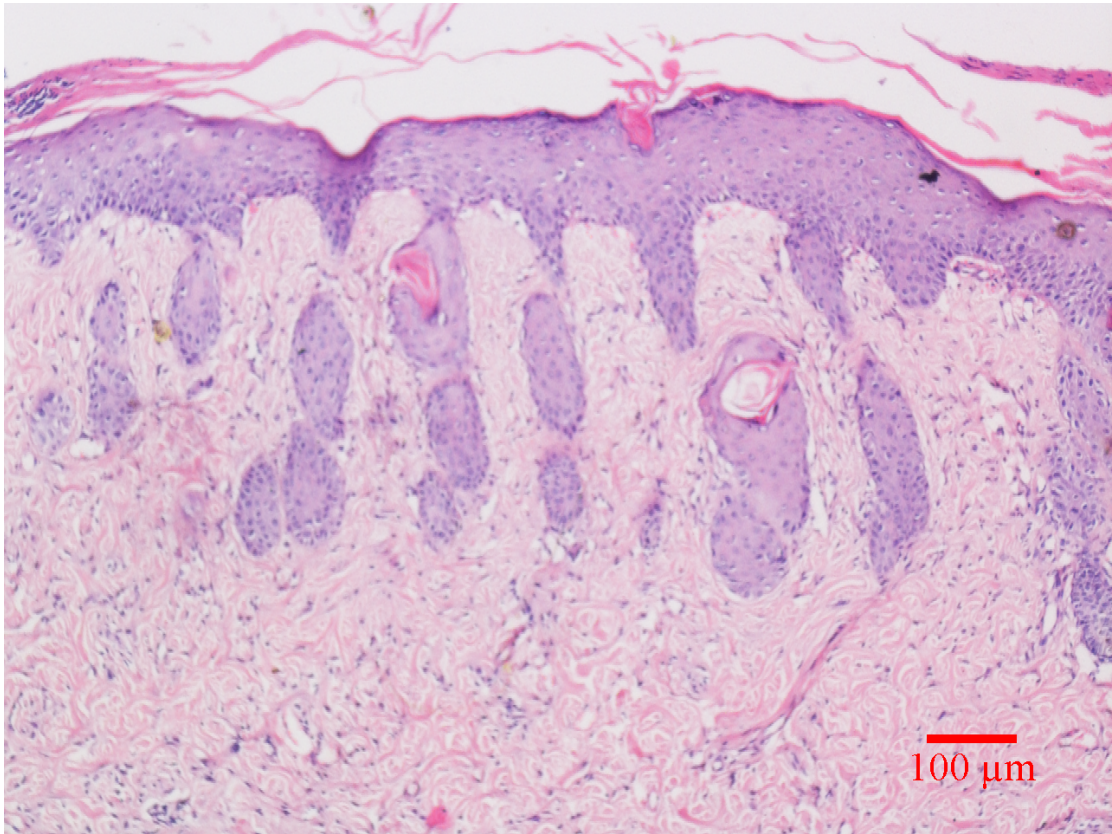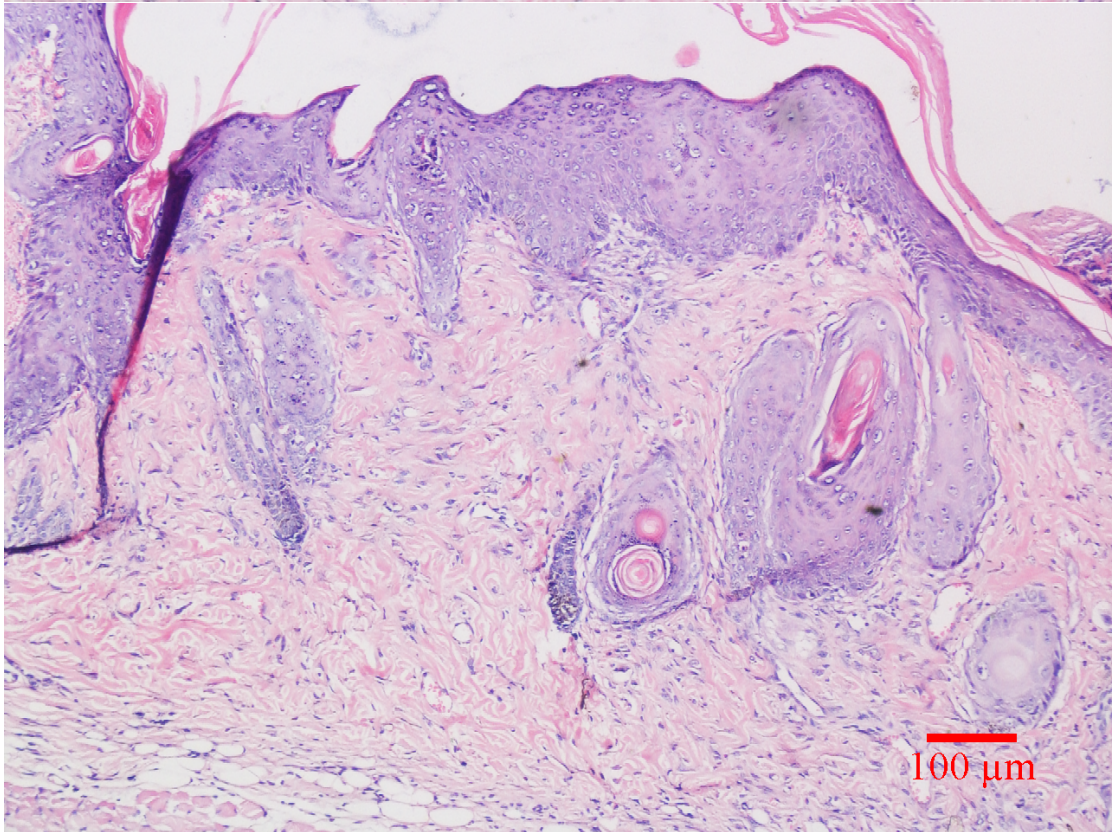

Supplement: Supplementary file 4 [file Image_1.pdf]
